# Predictors of in-hospital mortality after successful weaning of venoarterial extracorporeal membrane oxygenation in cardiogenic shock

**DOI:** 10.1038/s41598-023-44679-2

**Published:** 2023-10-16

**Authors:** Joo Hee Jeong, Hyungdon Kook, Seung Hun Lee, Hyung Joon Joo, Jae Hyoung Park, Soon Jun Hong, Mi-Na Kim, Seong-Mi Park, Jae Seung Jung, Jeong Hoon Yang, Hyeon-Cheol Gwon, Chul-Min Ahn, Woo Jin Jang, Hyun-Joong Kim, Jang-Whan Bae, Sung Uk Kwon, Wang Soo Lee, Jin-Ok Jeong, Sang-Don Park, Seong-Hoon Lim, Cheol Woong Yu

**Affiliations:** 1 Division of Cardiology, Department of Internal Medicine, Korea University College of Medicine and Korea University Anam Hospital, Goryeodae-ro, Sungbuk-ku, Seoul, 02841 Korea; 2 Division of Cardiology, Department of Internal Medicine, College of Medicine, Hanyang University, Seoul, Korea; 3 Division of Cardiology, Department of Internal Medicine, Donggunsan Hospital, Gunsan, Korea; 4 Department of Thoracic and Cardiovascular Surgery, Korea University College of Medicine and Korea University Anam Hospital, Seoul, Korea; 5 Division of Cardiology, Department of Medicine, Heart Vascular Stroke Institute, Samsung Medical Center, Sungkyunkwan University School of Medicine, Seoul, Korea; 6 Division of Cardiology, Severance Cardiovascular Hospital, Yonsei University College of Medicine, Seoul, South Korea; 7 Department of Cardiology, Ewha Woman’s University Seoul Hospital, Ehwa Woman’s University School of Medicine, Seoul, Korea; 8 Division of Cardiology, Department of Medicine, Konkuk University Medical Center, Seoul, Korea; 9 Department of Internal Medicine, Chungbuk National University College of Medicine, Cheongju, Korea; 10 Division of Cardiology, Department of Internal Medicine, Ilsan Paik Hospital, Inje University College of Medicine, Goyang, Korea; 11 Division of Cardiology, Department of Medicine, Chung-Ang University Hospital, Seoul, Korea; 12 Division of Cardiology, Department of Internal Medicine, Chungnam National University Hospital, Daejeon, Korea; 13 Division of Cardiology, Department of Medicine, Inha University Hospital, Incheon, Korea; 14 Division of Cardiovascular Medicine, Department of Internal Medicine, Dankook University Hospital, Dankook University College of Medicine, Cheonan, Korea

**Keywords:** Interventional cardiology, Cardiac device therapy

## Abstract

Limited knowledge exists regarding the predictors of mortality after successful weaning of venoarterial extracorporeal membrane oxygenation (ECMO). We aimed to identify predictors of in-hospital mortality in patients with cardiogenic shock (CS) after successful weaning from ECMO. Data were obtained from a multicenter registry of CS. Successful ECMO weaning was defined as survival with minimal mean arterial pressure (> 65 mmHg) for > 24 h after ECMO removal. The primary outcome was in-hospital mortality after successful ECMO weaning. Among 1247 patients with CS, 485 received ECMO, and 262 were successfully weaned from ECMO. In-hospital mortality occurred in 48 patients (18.3%). Survivors at discharge differed significantly from non-survivors in age, cardiovascular comorbidities, cause of CS, left ventricular ejection fraction, and use of adjunctive therapy. Five independent predictors for in-hospital mortality were identified: use of continuous renal replacement therapy (odds ratio 5.429, 95% confidence interval [CI] 2.468–11.940; p < 0.001), use of intra-aortic balloon pump (3.204, 1.105–9.287; p = 0.032), diabetes mellitus (3.152, 1.414–7.023; p = 0.005), age (1.050, 1.016–1.084; p = 0.003), and left ventricular ejection fraction after ECMO insertion (0.957, 0.927–0.987; p = 0.006). Even after successful weaning of ECMO, patients with irreversible risk factors should be recognized, and careful monitoring should be done for sign of deconditioning.

## Introduction

Cardiogenic shock (CS) is defined as a low cardiac output caused by various pump failure factors that leads to end-organ hypoperfusion and potentially life-threatening consequences. The clinical course of CS varies depending on its cause and severity. Extracorporeal membrane oxygenation (ECMO) plays a pivotal role in providing hemodynamic support for patients with CS through mechanical circulatory support, which alters the therapeutic strategies for CS. Although venoarterial ECMO provides powerful hemodynamic support to depressed myocardium, it mainly bridges recovery in most CS patients, serving as a short-term bridging mechanical circulatory support rather than a definitive therapy. After hemodynamic stabilization, promptly managing the primary cause of shock and establishing an appropriate off-pump time become crucial. Constant efforts have been made to determine the proper timing for successful weaning and removal of ECMO after overcoming shock. Although not fully established, several criteria and protocols for ECMO weaning considering hemodynamic and echocardiographic parameters have been suggested^1–4^.

However, recovery from CS and successful weaning from ECMO do not always guarantee favorable outcomes for all patients. Although several risk factors have been established to predict short-term mortality in patients with CS requiring ECMO support, limited knowledge exists regarding the outcomes of refractory CS after successful weaning and removal of ECMO^5–7^. Thus, we aimed to investigate the clinical characteristics of refractory CS after successful weaning of venoarterial ECMO and further assess the differential predictors of in-hospital mortality in this population.

## Methods

### Study design, setting, and participants

This study was based on the RESCUE registry (Retrospective and Prospective Observational Study to Investigate Clinical Outcomes and Efficacy of Left Ventricular Assist Device for Korean Patients with Cardiogenic Shock (NCT02985008 at http://www.clinicaltrials.gov). Twelve tertiary centers of South Korea participated in the enrollment of the RESCUE registry between January 2014 and December 2018. Adult patients (> 19-years old) were enrolled under the following inclusion criteria: (1) systolic blood pressure < 90 mmHg despite volume resuscitation or in need of inotropes, (2) sign of end organ hypoperfusion defined as cool extremity, oliguria (< 0.5 mL/kg per hour), altered mentality, lactate ≥ 2.0 mmol/L, or sign of pulmonary edema. Patients were excluded if: (1) shock occurred after out-of-hospital cardiac arrest, (2) evidence of shock of origin other than cardiogenic (hypovolemic, septic, or neurogenic) shock, or (3) they requested to discontinue participation in the study.

This study complied with the principles of the Declaration of Helsinki and was approved by the Institutional Review Board of each hospital. Detailed protocols and further information regarding data collection for the RESCUE registry have been previously published^8^.

### Patient management, outcome measurement and definition of variables

Venoarterial ECMO was performed at the physician’s discretion for short-term mechanical circulatory support of refractory CS. ECMO was removed (i) when the patient had successfully recovered from the critical phase of CS, or (ii) when it was deemed by a physician and legal representative that the patient was unlikely to recover from shock (hopeless removal). ECMO was weaned and removed with comprehensive consideration of clinical criteria, and successful ECMO weaning was defined as survival with minimal mean arterial pressure (> 65 mmHg) for more than 24 h after ECMO removal. The primary outcome was in-hospital mortality after successful weaning from ECMO, while the secondary outcome was all-cause mortality at the 1-year follow-up. The maximal usage of vasoactive agents was quantified with the inotropic and vasoactive-inotropic scores using the formula suggested by Gaies et al.^9^. Left ventricular ejection fraction (LV EF) was measured at the presentation of shock and was followed up upon clinical need. LV EF was specified by lowest LV EF before and after ECMO insertion. Further clinical definitions for each variable are provided in the supplemental material (Supplementary Table S1).

### Statistical analysis

Categorical variables were described as numbers and percentages, and continuous variables were described as means and standard deviations. To compare variables, the Student’s *t*-test, Mann–Whitney *U* test, chi-square test, or Fisher’s exact test was used as indicated. Logistic regression was used to identify the predictors of in-hospital mortality, and Kaplan–Meier analysis and the Cox proportional hazards model were used to assess time-dependent variables. Potential outliers and missing patterns of variables were examined, and variables with < 5% missing values were considered for the multivariable analysis (Supplementary Fig. S1). One variable (LV EF after ECMO insertion) revealed higher missing value, but was included for analysis regarding its clinical significance. Continuous variables with missing values were imputed using their mean value. Clinically intercorrelated variables were excluded from the multivariable regression analysis, and backward selection was used to identify statistically significant and 0.1 level for inclusion. Least Absolute Shrinkage and Selection Operator regression model was used to evaluate the coefficient of selected variables. Optimal lambda value that minimizes mean squared error was used to calculate coefficient. All tests were two-tailed, and statistical significance was defined as p values ≤ 0.05. All statistical analyses and model development were performed using the SPSS software (version 26; SPSS Inc., Chicago, IL, USA) and R Statistical software (version 4.2.3; R Foundation for Statistical Computing, Vienna, Austria).

### Ethics approval and consent to participate

The current study was approved by the Institutional Review Board of all participating hospitals (Korea University Anam Hospital, Samsung Medical Center, Severance Cardiovascular Hospital, Ewha Woman's University Seoul Hospital, Konkuk University Medical Center, Chungbuk National University College of Medicine, Ilsan Paik Hospital, Chung-Ang University Hospital, Chungnam National University Hospital, Inha University Hospital, Dankook University Hospital). The ethical guidelines of the 2013 Declaration of Helsinki and legal medical regulations of Republic of Korea were strictly undertaken throughout the study. Written informed consent was obtained from all participants or their legal representatives in patients that were prospectively enrolled, and written infomed consent was waived in the retrospectively enrolled patients.

## Results

### Study population

**Figure 1:**
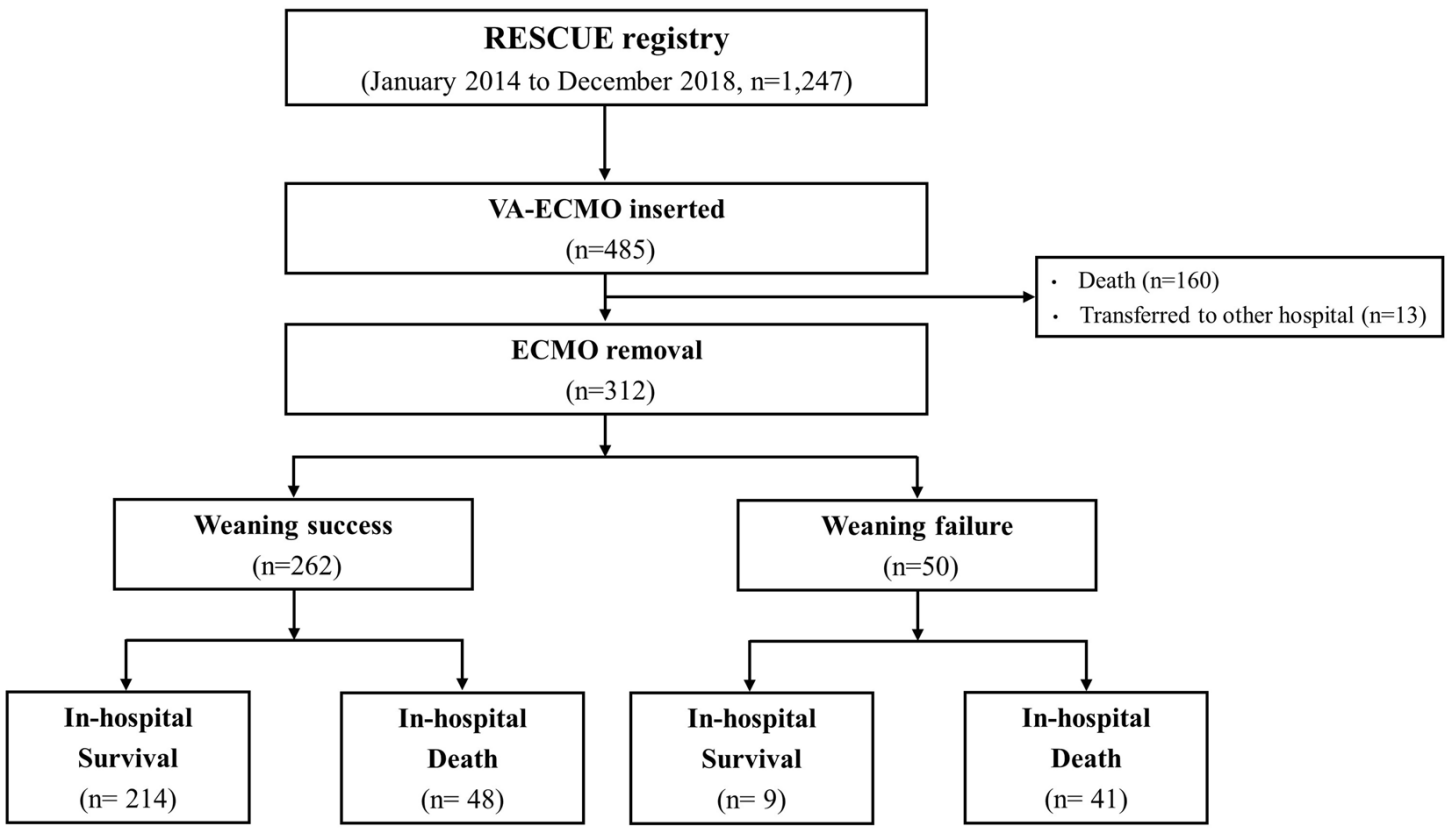
Flowchart of study. Among 1247 patients with cardiogenic shock, 485 received VA-ECMO support. Consecutive 312 patients were removed from ECMO, either after recovery from shock or after hopeless removal. Among patients who underwent ECMO removal (n = 312), 262 were successfully weaned by maintaining blood pressure (> 65 mmHg) for > 24 h after ECMO removal. *ECMO* extracorporeal membrane oxygenation, *VA-ECMO* venoarterial extracorporeal membrane oxygenation.

A total of 1247 patients with CS were included in the RESCUE registry from 2014 to 2018. Among them, 485 underwent venoarterial ECMO for refractory CS (Fig. 1). Of these, 173 patients did not survive or were transferred to another hospital before ECMO removal. Consequently, 312 patients underwent ECMO removal, either after recovery from shock or after hopeless removal. ECMO was successfully weaned in 262 patients, whereas 50 failed to wean. Among 262 patients with successful ECMO weaning, 48 (18.3%) did not survive to discharge.

Baseline characteristics of the 262 consecutive patients with successful ECMO weaning are shown in Table 1. Overall, 182 patients (69.5%) were male, and the age of onset was 60.1 ± 14.1 years. Additionally, 183 patients (69.8%) experienced ischemic CS, of which 95 (36.3%) presented with ST-elevation myocardial infarction. Adjunctive therapies were applied in addition to ECMO: intra-aortic balloon pump (IABP) in 28 patients (10.7%), continuous renal replacement therapy (CRRT) in 75 patients (28.6%), and mechanical ventilation in 192 patients (73.3%). The mean durations of intensive care unit and hospital stays were 23.6 ± 30.4 days and 40.3 ± 40.9 days, respectively.

**Table 1:** Baseline characteristics of patients with CS with successful ECMO weaning.

Variables	Total (n = 262)	Survivor (n = 214)	Non-survivor (n = 48)	t-value	p-value
Age (years)	60.1 ± 14.1	58.7 ± 13.8	66.6 ± 14.0	− 3.566	< 0.001
Male sex	182 (69.5)	147 (68.7)	35 (72.9)	− 0.573	0.567
Body mass index (kg/m^2^)	23.2 ± 3.3	23.1 ± 3.4	23.5 ± 3.0	− 0.639	0.523
Current smoker	74 (28.2)	59 (27.6)	15 (31.3)	− 0.510	0.610
Medical history					
Hypertension	116 (44.3)	86 (40.2)	30 (62.5)	− 2.845	0.005
Diabetes mellitus	99 (37.8)	72 (33.6)	27 (56.3)	− 2.852	0.006
Dyslipidemia	59 (22.5)	39 (18.2)	20 (41.7)	− 3.059	0.003
Chronic kidney disease	15 (5.7)	8 (3.7)	7 (14.6)	− 2.042	0.046
Previous myocardial infarction	30 (11.5)	21 (9.8)	9 (18.8)	− 1.478	0.145
Previous PCI	38 (14.5)	31 (14.5)	7 (14.6)	− 0.017	0.986
Previous CABG	5 (1.9)	4 (1.9)	1 (2.1)	− 0.098	0.922
Previous cerebrovascular accident	22 (8.4)	16 (7.5)	6 (12.5)	− 0.975	0.333
Previous CPR	15 (5.7)	14 (6.5)	1 (2.1)	1.660	0.099
Systolic blood pressure (mmHg)	68.6 ± 30.0	70.0 ± 30.4	62.6 ± 27.5	1.545	0.124
Diastolic blood pressure (mmHg)	46.5 ± 21.5	47.6 ± 22.1	41.7 ± 18.3	1.698	0.091
Heart rate	87.4 ± 37.1	88.7 ± 35.4	81.8 ± 43.9	1.012	0.316
Inotropic score	17.6 ± 24.6	16.0 ± 22.8	24.7 ± 31.0	− 2.233	0.026
Vasoactive-inotropic score	72.9 ± 163.3	69.5 ± 173.9	88.2 ± 103.3	− 0.713	0.476
Ischemic cardiogenic shock	183 (69.8)	142 (66.4)	41 (85.4)	− 3.134	0.002
STEMI	95 (36.3)	72 (33.6)	23 (47.9)	− 1.790	0.078
Initial laboratory markers					
Hemoglobin (g/dL)	12.4 ± 2.6	12.5 ± 2.7	12.3 ± 2.6	0.361	0.718
Platelet count (× 10^3^/μL)	203.5 ± 86.1	204.0 ± 89.1	201.1 ± 72.1	0.210	0.834
Creatinine (mg/dL)	1.4 ± 1.0	1.4 ± 1.0	1.7 ± 1.0	− 1.724	0.086
Lactic acid before ECMO insertion (mmol/L)	6.8 ± 4.1	6.7 ± 4.1	7.5 ± 4.1	− 1.188	0.236
Lactic acid after ECMO insertion (mmol/L)	2.7 ± 3.0	2.7 ± 3.0	3.0 ± 3.1	− 0.629	0.530
Peak troponin-I (ng/mL)	67.6 ± 151.4	58.0 ± 123.2	113.6 ± 241.4	− 1.471	0.148
Peak CK-MB (ng/mL)	177.0 ± 224.8	155.8 ± 200.5	274.0 ± 296.7	− 2.576	0.013
NT-proBNP (pg/mL)	9495.7 ± 13,660.9	9835.4 ± 14,172.2	7759.5 ± 10,727.4	0.721	0.472
LV EF before ECMO insertion (%)	28.7 ± 15.7	29.3 ± 16.3	26.2 ± 13.1	0.952	0.343
LV EF after ECMO insertion (%)	36.5 ± 16.8	38.6 ± 17.1	27.4 ± 11.6	2.397	< 0.001
Shock-to-ECMO insertion time (min)	289.1 ± 690.4	265.9 ± 649.4	394.6 ± 852.9	− 0.964	0.339
Adjunctive therapy					
Use of IABP	28 (10.7)	17 (7.9)	11 (22.9)	− 2.338	0.023
Use of CRRT	75 (28.6)	47 (22.0)	28 (58.3)	− 4.705	< 0.001
Use of mechanical ventilator	192 (73.3)	148 (69.2)	44 (91.7)	− 4.392	< 0.001
ECPR	101 (38.5)	74 (34.6)	27 (56.3)	− 2.819	0.005
ICU stay (day)	23.6 ± 30.4	24.3 ± 32.8	20.1 ± 15.8	1.297	0.197
Hospital stay (day)	40.3 ± 40.9	44.2 ± 43.4	23.2 ± 19.8	5.069	< 0.001

*CS* cardiogenic shock, *ECMO* extra-corporeal membrane oxygenation, *PCI* percutaneous coronary intervention, *CABG* coronary artery bypass graft, *CPR* cardiopulmonary resuscitation, *STEMI* ST-elevation myocardial infarction, *CK-MB* creatine kinase-MB, *NT-proBNP* N-terminal prohormone of brain natriuretic peptide, *LV EF* left ventricular ejection fraction, *IABP* intra-aortic balloon pump, *CRRT* continuous renal replacement therapy, *ECPR* extracorporeal cardiopulmonary resuscitation, *ICU* intensive care unit.

ECMO was primarily used as either a bridge to recovery (50.8%) or for decisions (24.8%, Supplementary Table S2). In majority of patients, ECMO was inserted with fluoroscopic guidance (77.5%) or percutaneously inserted (82.8%). After ECMO insertion, most patients received appropriate anticoagulation (93.9%). The mean duration of ECMO maintenance was 5.7 ± 5.9 days, and the most common ECMO-related complication was ECMO site bleeding, which occurred in 30 patients (11.5%).

Forty-eight consecutive patients (18.3%) died after successful weaning of ECMO. Compared with survivors, non-survivors were older (66.6 ± 14.0 vs. 58.7 ± 13.8 years, p < 0.001), and presented a higher frequency of comorbidities, including hypertension (62.5 vs. 40.2%, p = 0.005), diabetes mellitus (56.3 vs. 33.6%, p = 0.006), dyslipidemia (41.7 vs. 18.2%, p = 0.003), and chronic kidney disease (14.5 vs. 3.7%, p = 0.046, Table 1). Non-survivors had a higher proportion of ischemic CS (85.4 vs. 66.4%, p = 0.002) and a higher incidence of extracorporeal cardiopulmonary resuscitation (56.3 vs. 34.6%, p = 0.005). Non-survivors also had a higher inotropic score (24.7 ± 31.0 vs. 16.0 ± 22.8, p = 0.026), higher peak creatine kinase-MB (CK-MB) level (274.0 ± 296.7 vs. 155.8 ± 200.5 ng/mL, p = 0.013), and higher usage of adjunctive therapies of IABP (22.9 vs. 7.9%, p = 0.023), CRRT (58.3 vs. 22.0%, p < 0.001), and mechanical ventilation (91.7 vs. 69.2%, p < 0.001). LV EF measured before ECMO insertion did not differ between two groups (26.2 ± 13.1 vs. 29.3 ± 16.3%, p = 0.343), but showed significant difference after ECMO insertion (27.4 ± 11.6 vs. 38.6 ± 17.1%, p < 0.001). Notably, other parameters representing the severity of the early phase of shock (blood pressure, initial laboratory markers other than peak CK-MB, shock-to-ECMO insertion time, and initial pump flow) did not significantly differ between survivors and non-survivors.

### Predictors for in-hospital mortality

**Figure 2:**
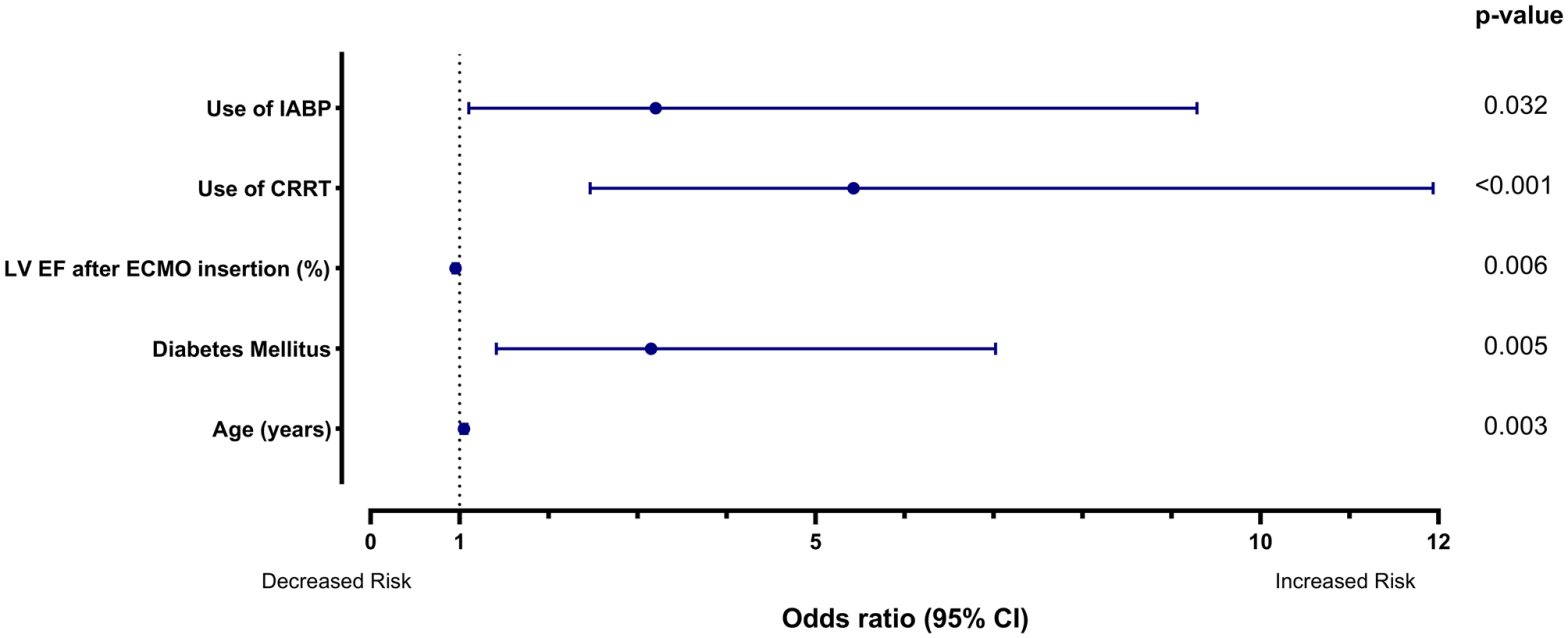
Predictors of in-hospital mortality. *IABP* intra-aortic balloon pump, *CRRT* continuous renal replacement therapy, *LV EF* left ventricular ejection fraction, *ECMO* extracorporeal membrane oxygenation, *CI* confidence interval.

Predictors of in-hospital mortality were identified using multivariable logistic regression analysis. Statistically significant variables in the univariable logistic regression analysis were as follows: age, hypertension, diabetes mellitus, dyslipidemia, chronic kidney disease, inotropic score, ischemic origin, peak CK-MB level, LV EF after ECMO insertion, use of IABP, use of CRRT, use of a mechanical ventilator, and extracorporeal cardiopulmonary resuscitation (Supplementary Table S3). In multivariable regression analysis, use of CRRT was the strongest predictor of in-hospital mortality (odds ratio [OR] 5.429, 95% confidence interval [CI] 2.468–11.940; p < 0.001; Table 2, Fig. 2). Other independent predictors included the use of IABP (OR 3.204, 95% CI 1.105–9.287; p = 0.032), diabetes mellitus (OR 3.152, 95% CI 1.414–7.023; p = 0.005), age (OR 1.050, 95% CI 1.016–1.084; p = 0.003), and LV EF after ECMO insertion (OR 0.957, 95% CI 0.927–0.987; p = 0.006). Least Absolute Shrinkage and Selection Operator regression analysis revealed highest coefficient in use of CRRT, followed bv use of IABP (Supplementary Table S4).

**Table 2:** Predictors of in-hospital mortality.

	Multivariable logistic regression analysis		
	Odds ratio	95% confidence interval	p-value
Male sex	0.660	0.265–1.645	0.373
Age (years)	1.050	1.016–1.084	0.003
Body mass index (kg/m^2^)	0.988	0.872–1.118	0.845
Hypertension	1.646	0.670–4.044	0.277
Diabetes mellitus	3.152	1.414–7.023	0.005
Dyslipidemia	1.868	0.804–4.343	0.146
Chronic kidney disease	1.431	0.339–6.046	0.626
Diastolic blood pressure	0.981	0.963–1.000	0.055
Inotropic score	1.000	0.987–1.014	0.978
Ischemic cardiogenic shock	1.045	0.337–3.244	0.939
Peak CK-MB (ng/mL)	1.002	1.000–1.003	0.060
LV EF after ECMO insertion (%)	0.957	0.927–0.987	0.006
Shock-to-ECMO insertion time (min)	1.000	1.000–1.001	0.261
Use of IABP	3.204	1.105–9.287	0.032
Use of CRRT	5.429	2.468–11.940	< 0.001
Use of mechanical ventilator	2.723	0.846–8.763	0.093
ECPR	1.895	0.777–4.620	0.160

*CK-MB* creatine kinase-MB, *LVEF* left ventricular ejection fraction, *ECMO* extracorporeal membrane oxygenation, *IABP* intra-aortic balloon pump, *CRRT* continuous renal replacement therapy, *ECPR* extracorporeal cardiopulmonary resuscitation.

### Predictors for 1-year all-cause mortality

**Figure 3:**
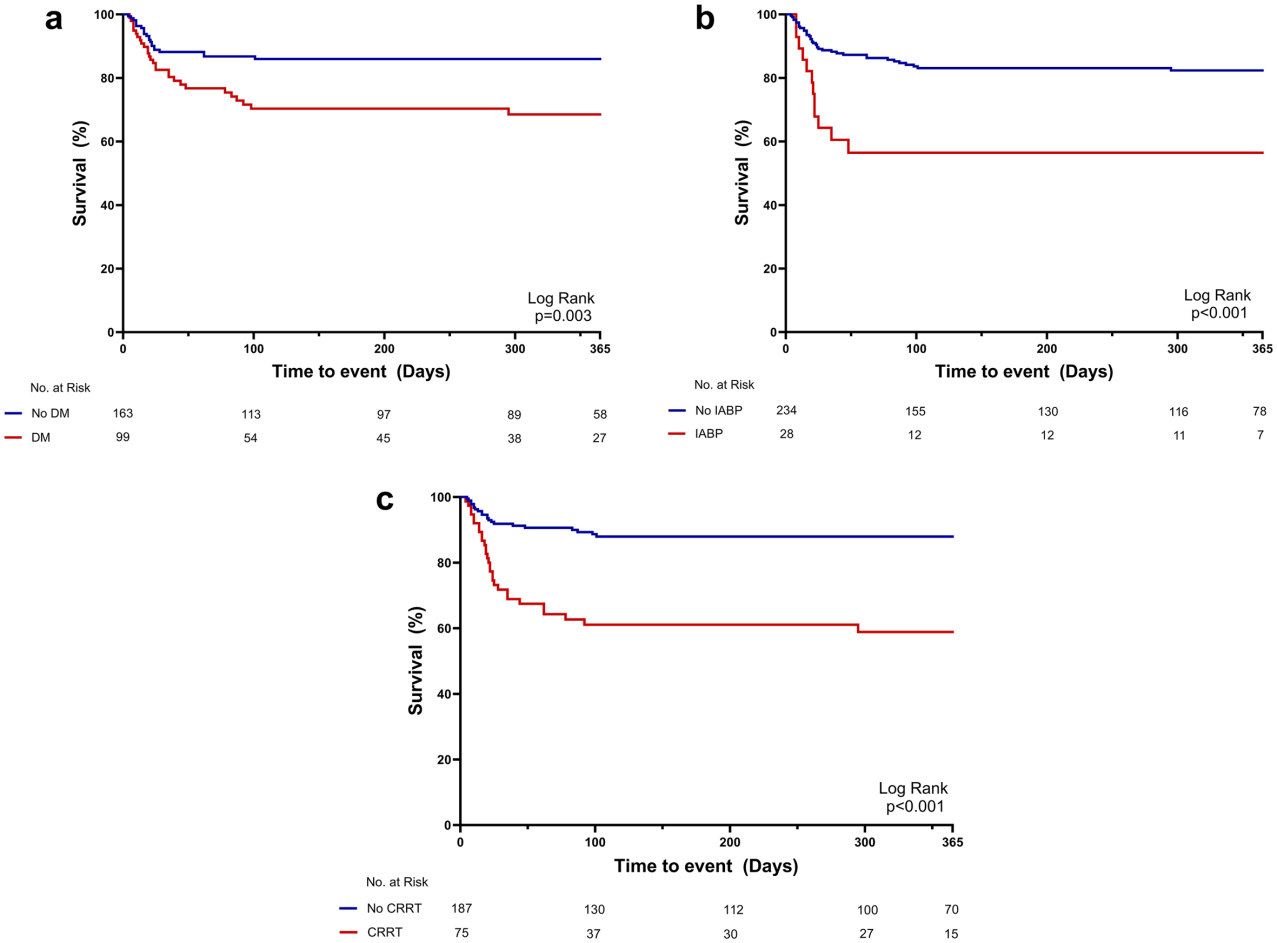
Kaplan–Meier curves for all-cause mortality at 1-year follow up (Binary variables). Kaplan–Meier curves of all-cause mortality according to presence of DM (**a**), use of IABP (**b**), and use of CRRT (**c**). *DM* diabetes mellitus, *IABP* intra-aortic balloon pump, *CRRT* continuous renal replacement therapy.

Patients who survived at discharge were followed up for one year. Patients without diabetes mellitus (Fig. 3a), IABP insertion (Fig. 3b), or CRRT (Fig. 3c) had a significantly higher survival rate during the follow-up. Furthermore, predictors of 1-year all-cause mortality were assessed using the Cox proportional hazards model (Supplementary Table S5). Independent predictors of 1-year mortality were mostly consistent with predictors of in-hospital mortality: use of CRRT (adjusted hazard ratio [aHR] 3.529, 95% CI 1.982–6.286; p < 0.001), use of IABP (aHR 2.365, 95% CI 1.164–4.804; p = 0.017), diabetes mellitus (aHR 2.361, 95% CI 1.300–4.289; p = 0.005), age (aHR 1.034, 95% CI 1.009–1.060; p = 0.007), peak CK-MB level (aHR 1.001, 95% CI 1.000–1.002; p = 0.008), and LV EF after ECMO insertion (aHR 0.965, 95% CI 0.943–0.988; p = 0.003, Table 3).

**Table 3:** Predictors of all-cause mortality at 1-year follow up.

	Multivariable Cox regression analysis		
	Adjusted hazard ratio	95% confidence interval	p-value
Male sex	0.703	0.354–1.394	0.313
Age (years)	1.034	1.009–1.060	0.007
Body mass index (kg/m^2^)	0.986	0.894–1.087	0.776
Hypertension	1.543	0.736–3.237	0.251
Diabetes mellitus	2.361	1.300–4.289	0.005
Dyslipidemia	1.545	0.825–2.893	0.174
Chronic kidney disease	1.135	0.415–3.102	0.806
Diastolic blood pressure	0.987	0.973–1.002	0.080
Inotropic score	1.002	0.992–1.011	0.720
Ischemic cardiogenic shock	1.151	0.462–2.867	0.763
Peak CK-MB (ng/mL)	1.001	1.000–1.002	0.008
LV EF after ECMO insertion (%)	0.965	0.943–0.988	0.003
Shock-to-ECMO insertion time (min)	1.000	1.000–1.001	0.127
Use of IABP	2.365	1.164–4.804	0.017
Use of CRRT	3.529	1.982–6.286	< 0.001
Use of mechanical ventilator	1.462	0.534–4.006	0.460
ECPR	1.647	0.922–2.940	0.092

*CK-MB* creatine kinase-MB, *LVEF* left ventricular ejection fraction, *ECMO* extracorporeal membrane oxygenation, *IABP* intra-aortic balloon pump, *CRRT* continuous renal replacement therapy, *ECPR* extracorporeal cardiopulmonary resuscitation.

Subgroups of non-survivors who succeeded in ECMO weaning and those who failed were further compared in terms of clinical characteristics (Supplementary Table S6). Several parameters that represent hypoperfusion in the initial phase of shock (vasoactive-inotropic score, lactic acid level after ECMO insertion) and comorbidities (diabetes mellitus, dyslipidemia) differed between the two groups, but the aforementioned predictors of mortality after successful ECMO weaning (age, LV EF after ECMO insertion, use of IABP, and use of CRRT) did not differ significantly.

## Discussion

This study investigated the clinical characteristics of patients who underwent successful ECMO weaning and identified the predictors of in-hospital mortality in this population. Although in-hospital mortality of patients with successful ECMO weaning was relatively low, it was not negligible (18.3%). Moreover, patients with successful ECMO weaning demonstrated distinct clinical characteristics compared with those who died in the early phase of CS. Namely, clinical parameters reflecting the severity of the early phase of CS were not identified as predictors of in-hospital mortality after successful weaning from ECMO. Predictors in this population underscored the significance of (i) risk factors that were not modifiable by ECMO support (age, pre-existing diabetes mellitus, prolonged pump failure measured as LV EF after ECMO insertion) and (ii) the need for adjunctive end-organ support even after ECMO insertion (IABP, CRRT).

Venoarterial ECMO has emerged as a salvage strategy for refractory CS that provides short-term cardiopulmonary support for patients with CS who do not respond to conventional medical therapies. Although the use of ECMO for refractory CS has increased over the past two decades, the outcome of refractory CS remains suboptimal^10^. Successful weaning is conventionally defined as having no requirement for further mechanical circulatory support 30 days after ECMO removal^11, 12^. However, successful weaning is not always equivalent to successful hospital discharge and survival^11^. Therefore, the procedure does not guarantee promising outcomes following ECMO device removal. As a result, constant efforts are needed to extend successful weaning to survival after discharge and long-term survival after discharge. The aforementioned clinical differences in this population underpin the different approaches to risk assessment in patients who have undergone successful ECMO weaning.

Several risk prediction scores have been developed to assess short-term mortality in patients with CS requiring ECMO^5, 13–15^. However, previous studies focused on the outcomes of ECMO-treated patients with CS as a whole, which comprised patients with two distinct clinical courses: those who did not survive until ECMO weaning and those who survived after ECMO weaning. While it is not fully established, distinct differences exist between patients who died in the early phase of CS and those who survived until ECMO weaning. Thus, previous risk scores, while useful for providing a generalized risk assessment for ECMO-treated patients with CS, may not be suitable for predicting outcomes in patients who survive until ECMO weaning.

### Comparison with previous studies of predictors of in-hospital mortality

Several studies on CS have identified the risk factors for in-hospital mortality. Schmidt et al. identified pre-ECMO factors that predict in-hospital survival and developed a risk calculation score called the survival after venoarterial ECMO score^5^. The independent predictors of in-hospital survival included demographic factors (age and weight), cause of CS, presence of organ failure, and several parameters assessed during the early phase of CS (pre-ECMO cardiac arrest, diastolic blood pressure before ECMO, peak inspiratory pressure, serum bicarbonate level, and duration of intubation before ECMO). Previous studies from the RESCUE registry reported similar findings. Yang et al. reported that age, body mass index, cardiac arrest at presentation, vasoactive-inotropic score, use of CRRT, use of a mechanical ventilator, use of IABP, and use of ECMO were independent predictors of in-hospital mortality for CS^8^. Seong et al. focused on ECMO-treated populations from the same registry and suggested the following predictors of in-hospital mortality: body mass index, lactic acid level, shock-to-ECMO time, cardiopulmonary resuscitation, use of a mechanical ventilator, use of CRRT, LV EF, and distal limb perfusion^6^. Namely, previous studies have highlighted (i) parameters that indicate the severity of the initial phase of CS, (ii) presence of other organ failures, and (iii) patients’ underlying demographic risk factors (age, weight, and comorbidities) as risk factors for mortality from CS.

In this study, in-hospital mortality for patients who underwent successful ECMO weaning was improved significantly (18.3%) compared with that in previous studies in the same cohort (33.6% in all CS cohort^8^, and 52.2% in ECMO-treated patients with CS^6^) and other studies that reported in-hospital mortality after ECMO weaning as high as 30%^16, 17^. However, in-hospital mortality was not negligible even after successful ECMO weaning. Interestingly, parameters indicative of the early phase of CS that were significant in previous studies (lactic acid level, shock-to-ECMO time, initial cardiac arrest, blood pressure, and vasopressor requirement) did not predict in-hospital mortality in this specific group. This finding implies that the mortality of patients with more severe CS (such as lower blood pressure with higher vasopressor requirement, higher lactic acid level, prolonged shock time, and a history of cardiac arrest) is determined during the early phase of CS. Accordingly, the mortality of those who survive the early phase of shock and proceed to successful ECMO weaning is not determined by early parameters. Rather, patients who die even after successful weaning of ECMO have significant risk factors, such as age, diabetes mellitus, or prolonged pump failure, that are not modifiable with ECMO support. In addition, these patients require adjunctive therapies in addition to ECMO, such as IABP and CRRT. A comparison of non-survivors who underwent successful ECMO weaning and those who did not (Supplementary Table S3) supports the robustness of the aforementioned predictors.

### Clinical implications

Our findings have several implications in clinical practice. In patients with CS who survive until successful weaning from venoarterial ECMO, a non-negligible degree of in-hospital mortality should be perceived. That is, even after successful weaning of ECMO, approximately one out of five patients do not survive to discharge. Patients with higher risk of in-hospital mortality after successful ECMO weaning—including those with older age, presence of diabetes mellitus, prolonged pump failure, history of using IABP or CRRT—should be recognized, and be informed of their risk. Consequently, more thorough monitoring should be done in this population to detect sign of deterioration, and prompt clinical intervention should be pursued. Careful monitoring should be maintained until successful discharge and follow-up after discharge.

Although we have identified extensive organ failure as a predictor for in-hospital mortality, specific risk factors that contribute to extensive organ failure need to be clarified. Ischemic CS is the most common cause of CS, which is known to have worse prognosis^18^. In our cohort, 85.4% of non-survivors had CS of ischemic origin. Coronary hypoperfusion with myocardial ischemia could contribute to multi-organ failure, and degree of coronary hypoperfusion is highly influenced by factors such as severity of coronary artery disease, time to revascularization, and revascularization strategy (culprit-only revascularization or multi-vessel revascularization)^19, 20^. Therefore, focusing on ischemic CS and identifying contributors to the myocardial ischemia may verify novel, modifiable risk factors that contributes to in-hospital mortality after successful weaning of ECMO.

### Limitations

This study had several limitations. First, although we specified successful weaning of ECMO as maintaining blood pressure after ECMO removal, the detailed reasons for weaning were not obtained. Thus, patients who underwent ECMO weaning after recovery from the shock phase were not distinguished from those who underwent unsuccessful ECMO removal. Consequently, patients with weaning failure (n = 50) may have included those who failed ECMO weaning after recovery from shock and those who underwent hopeless removal of ECMO. Nonetheless, only nine patients from the weaning failure group (n = 50) survived until discharge, implying that the survival rate was much lower in the weaning failure group than in the successful weaning group. Second, some clinical parameters may vary greatly according to the phase of shock and the use of supportive therapy. For instance, the assessment of LV EF is highly influenced by inotropic agents as well as the use of ECMO and its setting. Laboratory marker levels also change dynamically during shock; therefore, several variables were specified according to the temporal period and severity (i.e., highest lactic acid level before and after ECMO insertion, and lowest LV EF before and after ECMO insertion). We also attempted to specify clinical variables that reflect the most severe phase of shock, such as the lowest blood pressure measured at the date of shock, highest dose of vasoactive agents at the initial phase of shock (inotropic score, vasoactive-inotropic score), and highest level of cardiac markers (peak troponin-I and peak CK-MB levels). Finally, although diabetes mellitus was identified as an independent risk factor for mortality, further clinical information about diabetes mellitus, such as duration of diabetes or severity (glycosylated hemoglobin type A1c level, diabetic complications, and need for insulin), was not provided in this study. A similar shortcoming was seen in other parameters, such as the use of CRRT-specific clinical data, indications for CRRT, and serial changes in creatinine levels. Further studies with more specific clinical data may provide additional supporting evidence for our findings.

## Conclusion

Distinctive clinical characteristics exist between patients who survived ECMO weaning and those who did not, thus requiring a different approach to risk assessment. Independent predictors of in-hospital mortality identified after successful ECMO weaning include underlying irreversible risk factors and the need for adjunctive therapy for organ failure. Even after successful weaning of ECMO, patients with higher risk of mortality—that have irreversible risk factors or history of adjunctive therapy for organ failure—should be recognized. In this population, signs of deconditioning should be promptly discerned, and further thorough assessment should be done till successful discharge.

### Supplementary Information

Supplementary Information.

## Data Availability

All data generated or analyzed during this study are included in this published article and its supplementary information files.

## Abbreviations

aHR: Adjusted hazard ratio
CRRT: Continuous renal replacement therapy
CI: Confidence interval
CK-MB: Creatine kinase-MB
CS: Cardiogenic shock
ECMO: Extracorporeal membrane oxygenation
IABP: Intra-aortic balloon pump
LV EF: Left ventricular ejection fraction
OR: Odds ratio
