# Supplementary material for: Predictors of in-hospital mortality after successful weaning of venoarterial extracorporeal membrane oxygenation in cardiogenic shock
Source: Sci Rep. 2023 Oct 16;13:17529. doi: 10.1038/s41598-023-44679-2 (PMC10579350; doi:10.1038/s41598-023-44679-2)
Supplement: Supplementary file 1 — Supplementary Information. [file 41598_2023_44679_MOESM1_ESM.docx]

**SUPPLEMENTARY MATERIAL**

**Predictors of in-hospital mortality after successful weaning of venoarterial extracorporeal membrane oxygenation in cardiogenic shock**

**Running title:** Predicting mortality after venoarterial ECMO weaning

Joo Hee Jeong, MD^1†^, Hyungdon Kook, MD^2†^, Seung Hun Lee, MD^3^, Hyung Joon Joo, MD^1^, Jae Hyoung Park, MD^1^, Soon Jun Hong, MD^1^, Mi-Na Kim^1^, Seong-Mi Park^1^, Jae Seung Jung, MD^4^, Jeong Hoon Yang, MD^5^, Hyeon-Cheol Gwon, MD^5^, Chul-Min Ahn, MD^6^, Woo Jin Jang, MD^7^, Hyun-Joong Kim, MD^8^, Jang-Whan Bae, MD^9^, Sung Uk Kwon, MD^10^, Wang Soo Lee, MD^11^, Jin-Ok Jeong, MD^12^, Sang-Don Park, MD^13^, Seong-Hoon Lim, MD^14^, Cheol Woong Yu, MD^1^*****

**Supplementary Table S1. Definition of clinical variables**

| **Variables** | **Definition** |
| --- | --- |
| Age, years | Age at date of admission |
| BMI (kg/m^2^) | BMI at the date of admission |
| Current smoker | Current smoking or history of smoking within 3 months of admission |
| Hypertension | 1. Previously diagnosed hypertension, or 2. Blood pressure > 140/90 mmHg without vasoactive agents, or 3. On anti-hypertensive medications |
| Diabetes mellitus | 1. Previously diagnosed diabetes mellitus, or 2. Fasting glucose ≥ 126 mg/dL 3. HbA1c ≥ 6.5% |
| Dyslipidemia | 1. Previously diagnosed dyslipidemia, or 2. LDL-cholesterol > 160 mg/dL, or 3. Total cholesterol > 240 mg/dL |
| Chronic kidney disease | 1. Serum creatinine > 2.0 mg/dL without transient cause, or 2. History of kidney transplant, or 3. Undergoing renal replacement therapy |
| Previous myocardial infarction | 1. Previous medical record of myocardial infarction, or 2. Evident pathologic Q-wave at 12-lead electrocardiography (Q wave amplitude > 1/3 of R wave amplitude at two or more contiguous leads) |
| Previous cerebrovascular accident | Previous medical record of either ischemic or hemorrhagic stroke |
| Systolic blood pressure (mmHg) | The lowest systolic blood pressure measured at the date of shock |
| Diastolic blood pressure (mmHg) | The lowest diastolic blood pressure measured at the date of shock |
| Heart rate | The lowest heart rate measured at the date of shock |
| Ischemic cardiogenic shock | Clinical and angiographic evidence of myocardial ischemia that predispose to cardiogenic shock includes ST-elevation myocardial infarction, non-ST-elevation myocardial infarction, unstable angina, stable angina, variant angina, and ischemic cardiomyopathy |
| Inotropic score | Dopamine dose (μg/kg per minute) + dobutamine dose (μg/kg per minute) + 100×epinephrine dose (μg/kg per minute) |
| Vasoactive inotropic score | Dopamine dose (μg/kg per minute) + dobutamine dose (μg/kg per minute) + 100×epinephrine dose (μg/kg per minute) + 10×milrinone dose (μg/kg per minute) + 10,000×vasopressin dose (units/kg per minute) + 100×norepinephrine dose (μg/kg per minute). |
| Hb (g/dL) | The lowest Hb level measured at the date of shock before ECMO insertion |
| Platelet count (×10^3^/μL) | The lowest platelet count measured at the date of shock before ECMO insertion |
| Creatinine (mg/dL) | The highest creatinine level measured at the date of shock before ECMO insertion |
| Lactic acid before ECMO insertion | The highest lactic acid level measured at the date of shock before ECMO insertion |
| Lactic acid after ECMO insertion | The lowest lactic acid level measured during 24 h after ECMO insertion |
| Peak troponin-I (ng/mL) | The highest troponin-I level measured during admission |
| Peak CK-MB (ng/mL) | The highest CK-MB level measured during admission |
| NT-proBNP (pg/mL) | The highest NT-proBNP measured during admission |
| LV EF before ECMO insertion (%) | The lowest LV EF measured by echocardiography before ECMO insertion |
| LV EF after ECMO insertion (%) | The lowest LV EF measured during maintenance of ECMO and before weaning of ECMO, without changing ECMO flow for echocardiographic assessment |
| Shock-to-ECMO insertion time (min) | Time interval from the onset of shock to ECMO pump-on time |
| Use of IABP | Use of IABP at any time before or during ECMO insertion |
| Use of CRRT | Use of CRRT during ECMO treatment |
| Use of mechanical ventilator | Use of mechanical ventilator at the time of ECMO insertion |
| History of ECPR | An intention-to-treat with hemodynamic ECMO support during cardiac massage regardless of interim return of spontaneous circulation |

BMI, body mass index; HbA1c, glycosylated hemoglobin type A1c; LDL, low density lipoprotein; ECMO, extracorporeal membrane oxygenation; ECPR, extracorporeal cardiopulmonary resuscitation; STEMI, ST-elevation myocardial infarction; CABG, coronary artery bypass graft; IABP, intra-aortic balloon pump; CRRT, continuous renal replacement therapy; LV EF, left ventricular ejection fraction; Hb, hemoglobin; CK-MB, creatine kinase-MB; NT-proBNP, N-terminal prohormone of brain natriuretic peptide.

**Supplementary Table S2. ECMO-related variables**

| **Variables** | **Total**  **(n=262)** | **Survivor**  **(n=214)** | **Non-survivor**  **(n=48)** | **t-value** | **p-value** |
| --- | --- | --- | --- | --- | --- |
| Purpose of ECMO insertion* |  |  |  |  |  |
| Bridge to recovery | 133 (50.8) | 102 (47.7) | 31 (64.6) | -2.177 | 0.033 |
| Bridge to revascularization | 41 (15.6) | 34 (15.9) | 7 (14.6) | 0.224 | 0.823 |
| Bridge to transplantation | 30 (11.5) | 30 (14.0) | 0 (0.0) | 5.893 | <0.001 |
| Bridge to decision | 65 (24.8) | 55 (25.7) | 10 (20.8) | 0.704 | 0.482 |
| ECMO insertion in cardiology department | 202 (77.1) | 168 (78.5) | 34 (70.8) | 1.065 | 0.291 |
| Fluoroscopy-guided insertion | 203 (77.5) | 171 (79.9) | 32 (66.7) | 1.788 | 0.079 |
| Percutaneous insertion | 217 (82.8) | 179 (83.6) | 38 (79.2) | 0.741 | 0.459 |
| Anticoagulation during ECMO | 246 (93.9) | 202 (94.4) | 44 (91.7) | 0.711 | 0.478 |
| Distal perfusion | 124 (47.3) | 100 (46.7) | 24 (5.0) | -0.409 | 0.683 |
| Initial pump flow | 2.9 ± 0.7 | 2.9 ± 0.7 | 2.9 ± 0.8 | 0.490 | 0.625 |
| Initial RPM | 2633.4 ± 629.9 | 2630.7 ± 637.7 | 2645.4 ± 600.7 | -0.143 | 0.887 |
| ECMO duration (days) | 5.7 ± 5.9 | 5.8 ± 6.4 | 4.9 ± 2.6 | 1.575 | 0.117 |
| Complication during ECMO |  |  |  |  |  |
| Limb ischemia | 15 (5.7) | 11 (5.1) | 4 (8.3) | -0.859 | 0.391 |
| ECMO site bleeding | 30 (11.5) | 22 (10.3) | 8 (16.7) | -1.097 | 0.277 |
| Stroke | 8 (3.1) | 4 (1.9) | 4 (8.3) | -1.563 | 0.124 |
| Gastrointestinal bleeding | 10 (3.8) | 8 (3.7) | 2 (4.2) | -0.139 | 0.889 |
| Sepsis | 11 (4.2) | 6 (2.8) | 5 (10.4) | -1.656 | 0.104 |

*Seven patients had more than one purpose for ECMO insertion.

ECMO, extracorporeal membrane oxygenation; RPM, rounds per minute.

**Supplementary Table S3. Univariable logistic regression analysis for in-hospital mortality**

|  | **Univariable** | | |
| --- | --- | --- | --- |
|  | **Odds ratio** | **95% confidence interval** | **p-value** |
| Male sex | 0.815 | 0.405 - 1.640 | 0.566 |
| Age | 0.957 | 0.933 - 0.981 | 0.001 |
| Body mass index | 0.970 | 0.885 - 1.064 | 0.525 |
| Hypertension | 0.403 | 0.211 - 0.768 | 0.006 |
| Diabetes mellitus | 0.394 | 0.209 - 0.746 | 0.004 |
| Dyslipidemia | 0.312 | 0.160 - 0.610 | 0.001 |
| Chronic kidney disease | 0.227 | 0.078 - 0.662 | 0.007 |
| Diastolic blood pressure | 1.013 | 0.998 - 1.029 | 0.089 |
| Inotropic score | 0.989 | 0.978 - 0.999 | 0.039 |
| Ischemic cardiogenic shock | 0.337 | 0.144 - 0.788 | 0.012 |
| Peak CK-MB | 0.998 | 0.997 - 0.999 | 0.003 |
| LV EF after ECMO insertion | 1.047 | 1.022 - 1.073 | <0.001 |
| Shock-to-ECMO insertion time | 1.000 | 0.999 - 1.000 | 0.264 |
| Use of IABP | 0.290 | 0.126 - 0.670 | 0.004 |
| Use of CRRT | 0.201 | 0.104 - 0.388 | <0.001 |
| Use of mechanical ventilator | 0.204 | 0.070 - 0.591 | 0.003 |
| ECPR | 0.411 | 0.218 - 0.777 | 0.006 |

CK-MB, creatine kinase-MB; LV EF, left ventricular ejection fraction; ECMO, extracorporeal membrane oxygenation; IABP, Intra-aortic balloon pump; CRRT, Continuous renal replacement therapy; ECPR, extracorporeal cardiopulmonary resuscitation.

**Supplementary Table S4. Coefficients of predictors with Lasso regression analysis**

|  | **Coefficient** |
| --- | --- |
| Intercept | 0.90726 |
| Male sex | 0.03156 |
| Age (Years) | -0.00299 |
| Body mass index (kg/m2) | 0.00555 |
| Hypertension | -0.04211 |
| Diabetes mellitus | -0.11569 |
| Dyslipidemia | -0.09120 |
| Chronic kidney disease | -0.08823 |
| Diastolic blood pressure | 0.00157 |
| Inotropic score | -0.00025 |
| Ischemic cardiogenic shock | 0.03968 |
| Peak CK-MB (ng/mL) | -0.00026 |
| LV EF after ECMO insertion (%) | 0.00305 |
| Shock-to-ECMO insertion time (min) | -0.00002 |
| Use of IABP | -0.14682 |
| Use of CRRT | -0.20226 |
| Use of mechanical ventilator | -0.03704 |
| ECPR | -0.07326 |

CK-MB, creatine kinase-MB; LV EF, left ventricular ejection fraction; ECMO, extracorporeal membrane oxygenation; IABP, Intra-aortic balloon pump; CRRT, Continuous renal replacement therapy; ECPR, extracorporeal cardiopulmonary resuscitation.

**Supplementary Table S5. Unadjusted Cox regression analysis for all-cause mortality**

|  | **Unadjusted** | | |
| --- | --- | --- | --- |
|  | **Hazard ratio** | **95% confidence interval** | **p-value** |
| Male sex | 1.145 | 0.619 - 2.118 | 0.666 |
| Age | 1.042 | 1.014 - 1.065 | <0.001 |
| Body mass index | 1.023 | 0.944 - 1.108 | 0.576 |
| Hypertension | 2.520 | 1.418 - 4.478 | 0.002 |
| Diabetes mellitus | 2.361 | 1.356 - 4.111 | 0.002 |
| Dyslipidemia | 2.779 | 1.590 - 4.857 | <0.001 |
| Chronic kidney disease | 3.202 | 1.504 - 6.814 | 0.003 |
| Diastolic blood pressure | 0.991 | 0.978 - 1.004 | 0.159 |
| Inotropic score | 1.009 | 1.002 - 1.016 | 0.011 |
| Ischemic cardiogenic shock | 3.007 | 1.354 - 6.678 | 0.007 |
| Peak CK-MB | 1.002 | 1.001 - 1.003 | <0.001 |
| LV EF after ECMO insertion | 0.962 | 0.942 - 0.982 | <0.001 |
| Shock-to-ECMO insertion time | 1.000 | 1.000 - 1.000 | 0.287 |
| Use of IABP | 3.053 | 1.596 - 5.840 | 0.001 |
| Use of CRRT | 3.763 | 2.160 - 6.555 | <0.001 |
| Use of mechanical ventilator | 3.594 | 1.428 - 9.047 | 0.007 |
| ECPR | 2.296 | 1.319 - 3.997 | 0.003 |

CK-MB, creatine kinase-MB; LV EF, left ventricular ejection fraction; ECMO, extracorporeal membrane oxygenation; IABP, Intra-aortic balloon pump; CRRT, Continuous renal replacement therapy; ECPR, extracorporeal cardiopulmonary resuscitation.

**Supplementary Table S6. Comparisons of subgroups regarding success of ECMO weaning**

| **Variables** | **No weaning success***  **(n=223)** | **Weaning success**  **(n=48)** | **t-value** | **p-value** |
| --- | --- | --- | --- | --- |
| Age, years | 63.6 ± 13.6 | 66.6 ± 14.0 | -1.358 | 0.175 |
| Male sex | 155 (69.5) | 35 (72.9) | -0.467 | 0.641 |
| Body mass index (kg/m^2^) | 23.4 ± 3.7 | 23.5 ± 3.0 | -0.137 | 0.891 |
| Current smoker | 60 (26.9) | 15 (31.3) | -0.608 | 0.543 |
| Medical history |  |  |  |  |
| Hypertension | 112 (50.2) | 30 (62.5) | -1.570 | 0.121 |
| Diabetes mellitus | 75 (33.6) | 27 (56.3) | -2.863 | 0.006 |
| Dyslipidemia | 42 (18.8) | 20 (41.7) | -2.863 | 0.004 |
| Chronic kidney disease | 23 (10.3) | 7 (14.6) | -0.853 | 0.394 |
| Previous myocardial infarction | 30 (13.5) | 9 (18.8) | -0.947 | 0.345 |
| Systolic blood pressure (mmHg) | 63.4 ± 33.2 | 62.6 ± 27.5 | 0.157 | 0.876 |
| Diastolic blood pressure (mmHg) | 40.5 ± 22.9 | 41.7 ± 18.3 | -0.359 | 0.720 |
| Heart rate | 77.7 ± 39.6 | 81.8 ± 43.9 | -0.630 | 0.529 |
| Inotropic score | 37.2 ± 54.3 | 24.7 ± 31.0 | 1.537 | 0.125 |
| Vasoactive inotropic score | 134.2 ± 149.1 | 88.2 ± 103.3 | 2.563 | 0.012 |
| Ischemic cardiogenic shock | 172 (77.1) | 41 (85.4) | -1.412 | 0.162 |
| Laboratory markers |  |  |  |  |
| Hemoglobin (g/dL) | 12.3 ± 2.9 | 12.3 ± 2.6 | 0.066 | 0.948 |
| Platelet count (×10^3^/μL) | 190.0 ± 83.7 | 201.1 ± 72.1 | -0.852 | 0.395 |
| Creatinine (mg/dL) | 1.7 ± 1.5 | 1.7 ± 1.0 | 0.075 | 0.940 |
| Lactic acid before ECMO insertion (mmol/L) | 8.9 ± 4.7 | 7.5 ± 4.1 | 1.653 | 0.100 |
| Lactic acid after ECMO insertion (mmol/L) | 7.3 ± 5.6 | 3.0 ± 3.1 | 6.554 | <0.001 |
| Peak troponin-I (ng/mL) | 62.4 ± 126.9 | 113.6 ± 241.4 | -1.353 | 0.183 |
| Peak CK-MB (ng/mL) | 271.6 ± 610.3 | 274.0 ± 296.7 | -0.026 | 0.979 |
| NT-proBNP (pg/mL) | 9234.3 ± 11182.2 | 7759.5 ± 10727.4 | 0.626 | 0.532 |
| LV EF before ECMO insertion (%) | 29.2 ± 16.4 | 25.7 ± 13.2 | 1.053 | 0.294 |
| LV EF after ECMO insertion (%) | 23.4 ± 14.4 | 27.4 ± 11.6 | -1.691 | 0.094 |
| Shock-to-ECMO insertion time (min) | 530.4 ± 966.6 | 394.6 ± 852.9 | 0.882 | 0.379 |
| Use of IABP | 34 (15.2) | 11 (22.9) | -1.164 | 0.249 |
| Use of CRRT | 117 (52.5) | 28 (58.3) | -0.737 | 0.462 |
| Use of mechanical ventilator | 209 (93.7) | 44 (91.7) | 0.517 | 0.606 |
| ECPR | 133 (59.6) | 27 (56.3) | 0.432 | 0.666 |
| Initial pump flow | 2.8 ± 0.9 | 2.9 ± 0.8 | -0.629 | 0.530 |
| Initial rotations per minute | 2593.1 ± 629.0 | 2645.4 ± 600.7 | -0.514 | 0.608 |
| ECMO duration (days) | 4.8 ± 5.0 | 4.8 ± 2.6 | -0.091 | 0.928 |

*This subgroup includes patients that received venoarterial-ECMO, but (i) did not proceed to ECMO removal (n=173), or (ii) failed ECMO weaning (n=50).

ECMO, extracorporeal membrane oxygenation; CK-MB, creatine kinase-MB; NT-proBNP, N-terminal prohormone of brain natriuretic peptide; LV EF, left ventricular ejection fraction; IABP, Intra-aortic balloon pump; CRRT, Continuous renal replacement therapy; ECPR, extracorporeal cardiopulmonary resuscitation.

**Supplementary Figure S1. Proportion of missing values in predictors**

**
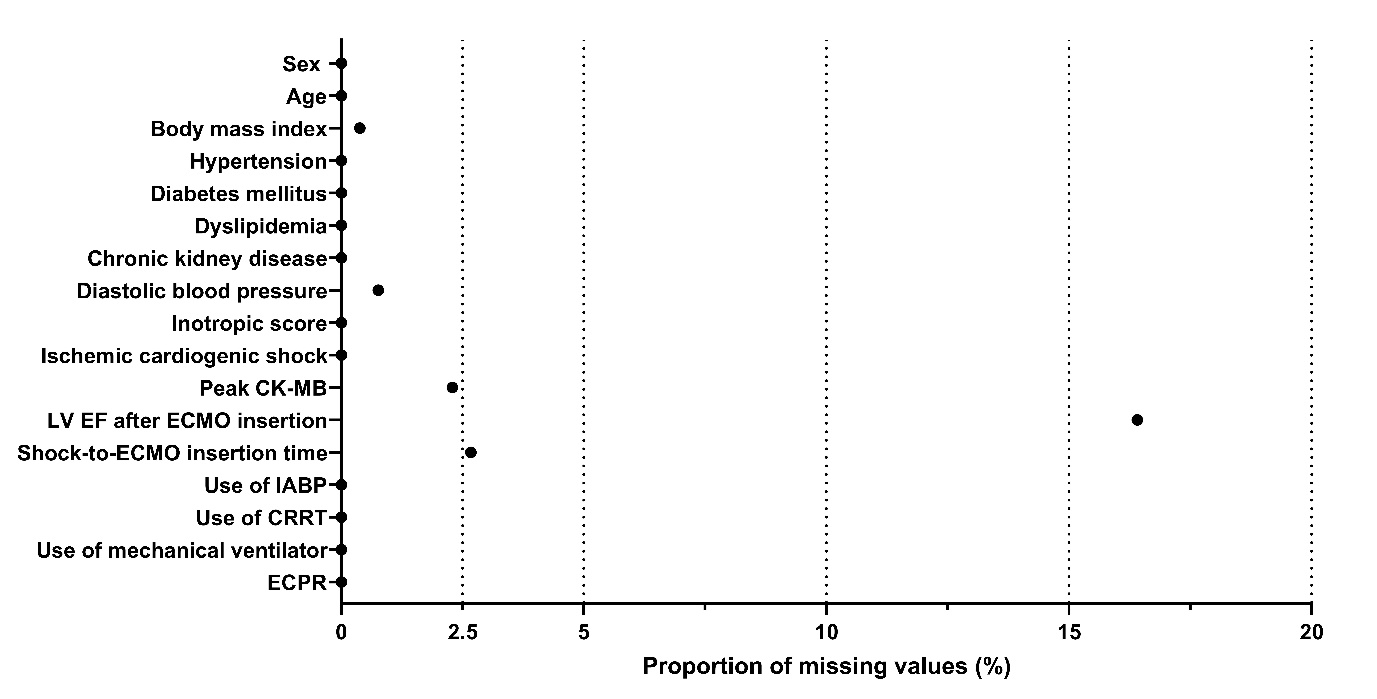
**

CK-MB, creatine kinase-MB; LV EF, left ventricular ejection fraction; ECMO, extracorporeal membrane oxygenation; IABP, Intra-aortic balloon pump; CRRT, Continuous renal replacement therapy; ECPR, extracorporeal cardiopulmonary resuscitation.
